# Combination Strategies to Improve Targeted Radionuclide Therapy

**DOI:** 10.2967/jnumed.120.248062

**Published:** 2020-11

**Authors:** Tiffany G. Chan, Edward O’Neill, Christine Habjan, Bart Cornelissen

**Affiliations:** Department of Oncology, University of Oxford, Oxford, United Kingdom

**Keywords:** targeted radionuclide therapy, combination therapy, radiotherapy

## Abstract

In recent years, targeted radionuclide therapy (TRT) has emerged as a promising strategy for cancer treatment. In contrast to conventional radiotherapy, TRT delivers ionizing radiation to tumors in a targeted manner, reducing the dose that healthy tissues are exposed to. Existing TRT strategies include the use of ^177^Lu-DOTATATE, ^131^I-metaiodobenzylguanidine, Bexxar, and Zevalin, clinically approved agents for the treatment of neuroendocrine tumors, neuroblastoma, and non-Hodgkin lymphoma, respectively. Although promising results have been obtained with these agents, clinical evidence acquired to date suggests that only a small percentage of patients achieves complete response. Consequently, there have been attempts to improve TRT outcomes through combinations with other therapeutic agents; such strategies include administering concurrent TRT and chemotherapy, and the use of TRT with known or putative radiosensitizers such as poly(adenosine diphosphate ribose) polymerase and mammalian-target-of-rapamycin inhibitors. In addition to potentially achieving greater therapeutic effects than the respective monotherapies, these strategies may lead to lower dosages or numbers of cycles required and, in turn, reduce unwanted toxicities. As of now, several clinical trials have been conducted to assess the benefits of TRT-based combination therapies, sometimes despite limited preclinical evidence being available in the public domain to support their use. Although some clinical trials have yielded promising results, others have shown no clear survival benefit from particular combination treatments. Here, we present a comprehensive review of combination strategies with TRT reported in the literature to date and evaluate their therapeutic potential.

**NOTEWORTHY**

- TRT is growing in popularity for cancer treatment.
- Efforts to improve TRT have led to increasing numbers of combination strategies being attempted.
- Increasing our understanding of the radiobiology of TRT will help inform the successful implementation of combination strategies.

Targeted radionuclide therapy (TRT) involves the use of radiopharmaceuticals designed to specifically target cancer cells. These radiopharmaceuticals consist of β, α, or Auger electron–emitting radionuclides coupled to a tumor-targeting vector, such as a monoclonal antibody or peptide. In recent years, TRT has rapidly grown in popularity, with agents such as ^177^Lu-DOTATATE,^131^I-metaiodobenzylguanidine, (Bexxar; GlaxoSmithKline), and (Zevalin; Acrotech Biopharma) now clinically approved for the treatment of low-grade neuroendocrine tumors (NETs), neuroblastoma, and non-Hodgkin lymphoma, respectively. ^177^Lu-PSMA, which targets prostate-specific membrane antigen (PSMA), is also emerging as an attractive strategy for metastatic castration-resistant prostate cancer, with large late-phase clinical trials under way. Because of its highly targeted approach, the application of TRT could result in fewer side effects than conventional external-beam radiotherapy (EBRT) and allow for more effective treatment of disseminated cancers (1). Promising results have been obtained with several TRTs in clinical trials, but there remains room for improvement; for instance, whereas the phase III NETTER-1 trial showed that ^177^Lu-DOTATATE can be a life-extending treatment, with significant improvement in the median progression-free survival being reported, objective response was observed in just 18% of patients (2). To further improve TRT outcomes, administering TRT at earlier disease stages, as is being investigated in the UpFrontPSMA trial (NCT04343885), and the use of combination therapies is being attempted.

Combination strategies investigated thus far include the use of agents to improve tumor perfusion to allow better distribution of the radiopharmaceutical (3), upregulation of the target receptor to increase cellular uptake (4), the combination of TRT with other DNA-damaging drugs (5–7), radiosensitization through inhibiting essential processes such as DNA damage repair (8–10), and the use of immune checkpoint inhibitors (11–13). Several of these strategies have shown promise in preclinical studies and are currently in clinical trials, but there remains a lack of research into TRT radiobiology, making it difficult to predict which of these strategies will prove most effective. Attempts to enhance tumor perfusion and cellular uptake have shown promise, but we do not currently know the exact absorbed radiation dose required for a complete response, making the optimization of this strategy challenging (3,4). Efforts toward modeling the necessary dose required for tumor response after ^177^Lu-PSMA therapy are under way, but this modeling is difficult to achieve given the heterogeneity of PSMA expression (14). Additionally, although there are a wide variety of drugs being combined with TRT in an attempt to achieve effective radiosensitization, the mechanisms by which these combination therapies work and their effects on downstream biologic pathways are not necessarily fully understood. Many of these combination strategies make use of known radiosensitizers of EBRT, but because of differences in the dose rates, duration of radiation exposure, and radiobiologic effects between EBRT and TRT, our understanding of EBRT radiobiology cannot simply be extrapolated to TRT (1,15–17). Radiosensitizers of EBRT may therefore not always be effective radiosensitizers of TRT, and vice versa. Here, we present a comprehensive review of TRT-based combination therapies tested to date, focusing primarily on those involving β-emitting TRTs because several of these combinations are being evaluated clinically (Fig. 1; Supplemental Tables 1–8 [supplemental materials are available at http://jnm.snmjournals.org]). We also discuss possible limitations of such studies and suggest further research that is needed if these combination strategies are to be effectively translated to patients.

**FIGURE 1.**
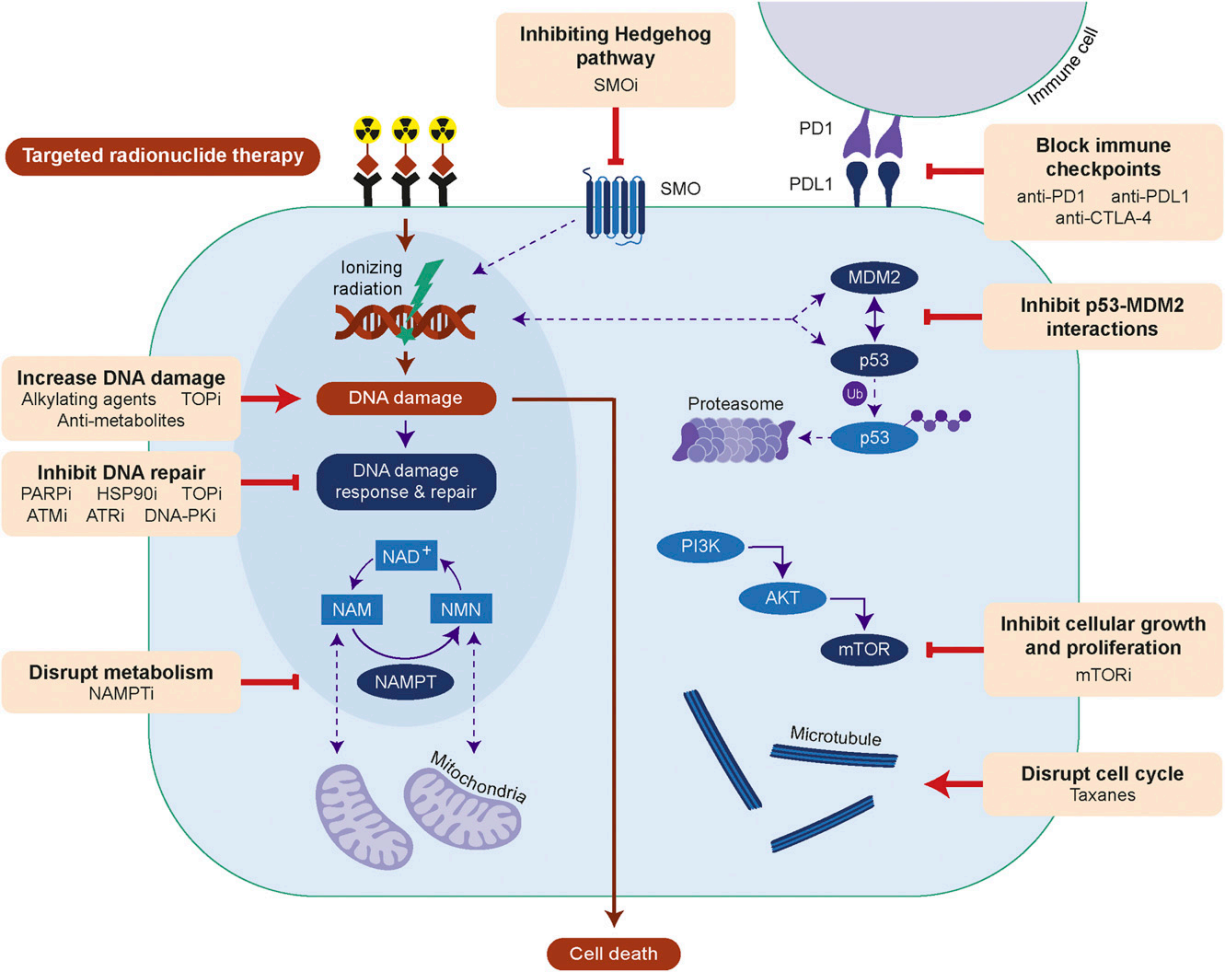
Efficacy of TRT can be improved by increasing DNA damage, inhibiting DNA repair, disrupting metabolism or cell cycle, inhibiting signaling of Hedgehog, phosphoinositide-3-kinase (PI3K)/protein kinase B (AKT)/mTOR or p53-MDM2, or blocking immune checkpoints. AKT = protein kinase B; ATMi = ataxia telangiectasia mutated inhibitor; ATRi = ataxia telangiectasia and Rad3-related inhibitor; CTLA-4 = cytotoxic T-lymphocyte antigen 4; HSP90i = HSP90 inhibitor; mTORi = mTOR inhibitor; NAM = nicotinamide; NAMPTi = NAMPT inhibitor; NMN = nicotinamide mononucleotide; PI3K = phosphoinositide-3-kinase; PARPi = PARP inhibitor; PD1 = programmed death 1; PDL1 = programmed death 1 ligand; PKi = protein kinase inhibitor; SMOi = smoothened inhibitor; SMO = smoothened; TOPi = topoisomerase inhibitor; Ub = ubiquitin.

## INCREASING DNA DAMAGE WITH TRADITIONAL CHEMOTHERAPIES

As TRT primarily acts by inducing DNA damage, the efficacy of TRT can be increased by creating additional DNA damage through combining TRT with conventional chemotherapy or radiotherapy. Prominent examples include the combination of ^177^Lu-DOTATATE with the antimetabolite capecitabine, the alkylating agent temozolomide, or both (CAPTEM) for the treatment of NETs (Supplemental Table 1). These are all standard chemotherapy options for advanced NETs, making their combination with ^177^Lu-DOTATATE easily translatable if effective. To date, promising tumor response rates have been observed with ^177^Lu-DOTATATE + capecitabine, ^177^Lu-DOTATATE + temozolomide, and/or ^177^Lu-DOTATATE + capecitabine + temozolomide (CAPTEM) (6,18,19), and phase II clinical trials are currently under way (NCT02736500 and NCT02358356). Initial results from the phase I/II CAPTEM trial suggest that although the objective tumor response rate in the cohort treated with ^177^Lu-DOTATATE + CAPTEM was higher than in patients treated with ^177^Lu-DOTATATE alone, more treatment-related adverse effects were observed (20). Though rare, both ^177^Lu-DOTATATE and CAPTEM have been associated with hematologic toxicities when used as monotherapies (2,21); thus, the increase in adverse events observed in the combination group may be due to overlapping toxicities. The final outcomes of the phase I/II CAPTEM trial should provide further insight.

**FIGURE 2.**
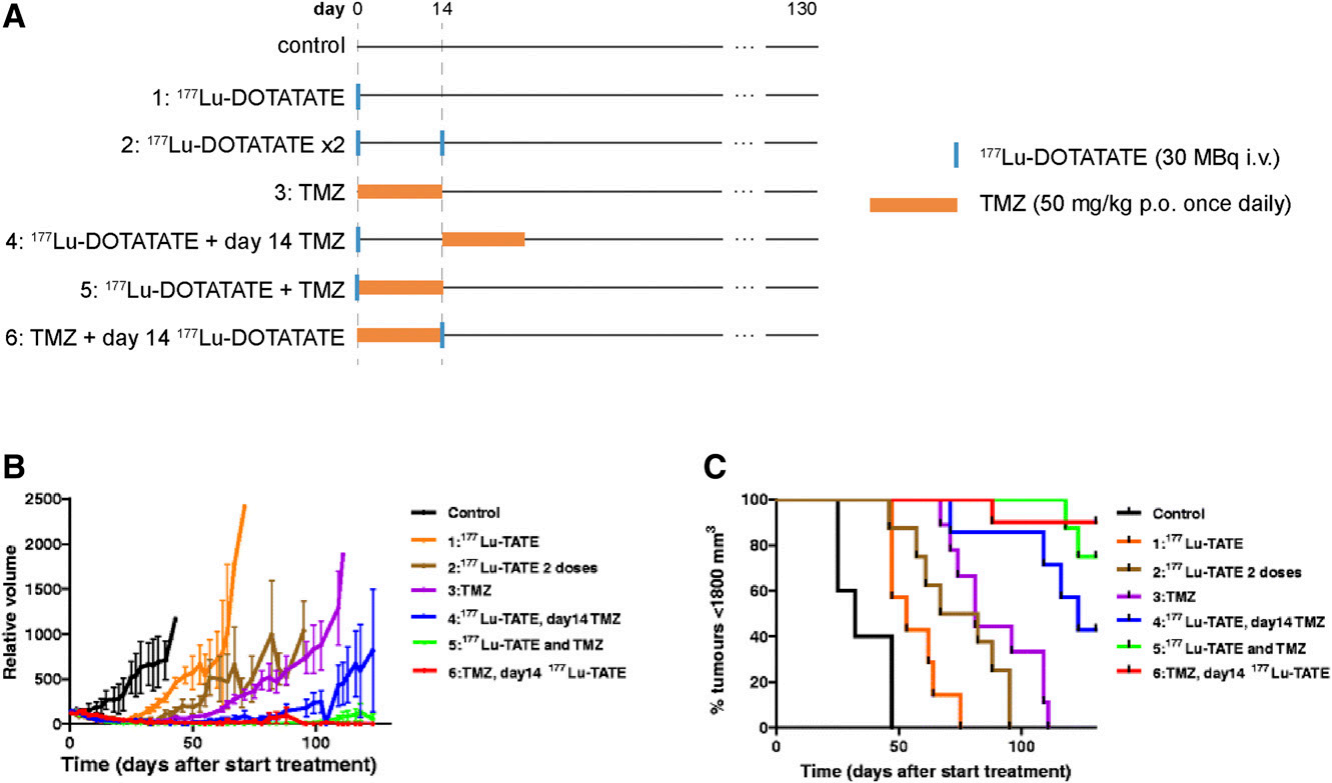
Evaluation of different ^177^Lu-DOTATATE + temozolomide (TMZ) treatments in H69 tumor–bearing mice. (A) Study timeline. (B and C) Average tumor volume and percentage of mice with tumors smaller than 1,800 mm^2^ (8–10 mice per group). i.v. = intravenously; p.o. = orally. (Reprinted with permission of (*3*).)

To our knowledge, there exists just one preclinical study attempting to optimize ^177^Lu-DOTATATE + capecitabine + temozolomide, ^177^Lu-DOTATATE + temozolomide chemotherapy (3). In mice bearing human small cell lung cancer H69 tumors, Bison et al. noted that although temozolomide outperformed ^177^Lu-DOTATATE as a monotherapy, the combination of ^177^Lu-DOTATATE + temozolomide led to additive effects (Fig. 2) (3). Moreover, administering temozolomide 14 d before ^177^Lu-DOTATATE treatment was found to be significantly more effective than vice versa, which was attributed to enhanced tumor perfusion, radiosensitivity, and tumor oxygenation (3). In clinical studies involving ^177^Lu-DOTATATE + temozolomide, ^177^Lu-DOTATATE + capecitabine + temozolomide therapy, chemotherapy is typically initiated before or concomitantly with ^177^Lu-DOTATATE (5,7,19). Further studies are necessary to optimize the dosing schedules to produce the greatest survival benefits, particularly for CAPTEM protocols, for which the relative timings of capecitabine and temozolomide administration are already known to affect synergism (22).

Aside from ^177^Lu-DOTATATE–based combination therapies, there are several examples of other TRTs being combined with chemotherapy, but there is currently insufficient evidence to support the use of these combinations over their respective monotherapies (Supplemental Table 1). Recent reports have, however, alluded to significantly longer overall survival times in chemotherapy-naïve patients treated with TRT than in patients with a history of chemotherapy (23–25). Until the precise reason behind these observations is understood, TRT + chemotherapy combinations should be carefully monitored if implemented.

## RADIOSENSITIZATION THROUGH INHIBITING DNA REPAIR

Inhibiting key proteins involved in the DNA damage response (DDR) could lead to radiosensitization of TRT. To date, there are several examples of this strategy being used: most involve poly(adenosine diphosphate ribose) polymerase (PARP) inhibitors, but inhibitors of other DDR proteins, heat shock protein 90 (HSP90), and checkpoint kinase 1 have also been used (Supplemental Table 2). Inhibition of DNA topoisomerases I and II has also been suggested to induce radiosensitization by disrupting DNA repair (15,16). It should be noted, however, that many DDR-targeting agents are associated with bone marrow and gastrointestinal toxicities, which could lead to increased adverse side effects when combined with TRT (2,26,27). Careful monitoring of normal-tissue toxicity is therefore warranted for these combinations.

### DDR Inhibitors

PARP enzymes constitute a family of proteins that are vital for the maintenance of cellular homeostasis and form an essential part of DDR. PARP proteins—in particular, PARP-1—have been implicated in the repair of both single-stranded and double-stranded breaks (28). Because the main mechanism by which ionizing radiation causes cell death is through the induction of DNA damage, reducing the capacity for DNA repair with PARP inhibitors is a potential radiosensitization strategy (29,30). Several preclinical studies investigated whether PARP inhibitors can also potentiate TRT with ^177^Lu-DOTATATE: in vitro with the human cancer cell lines osteosarcoma U2OS expressing somatostatin receptor 2, gastroenteropancreatic BON-1, and bronchopulmonary NCI-H727, and in vivo in AR42J tumor–bearing mice (8–10). The combination of PARP inhibitors with other TRTs, such as ^131^I-MIP-1095, ^177^Lu-RM2, and ^227^Th-HER2, has also been tested, with promising results (Supplemental Table 2).

**FIGURE 3.**
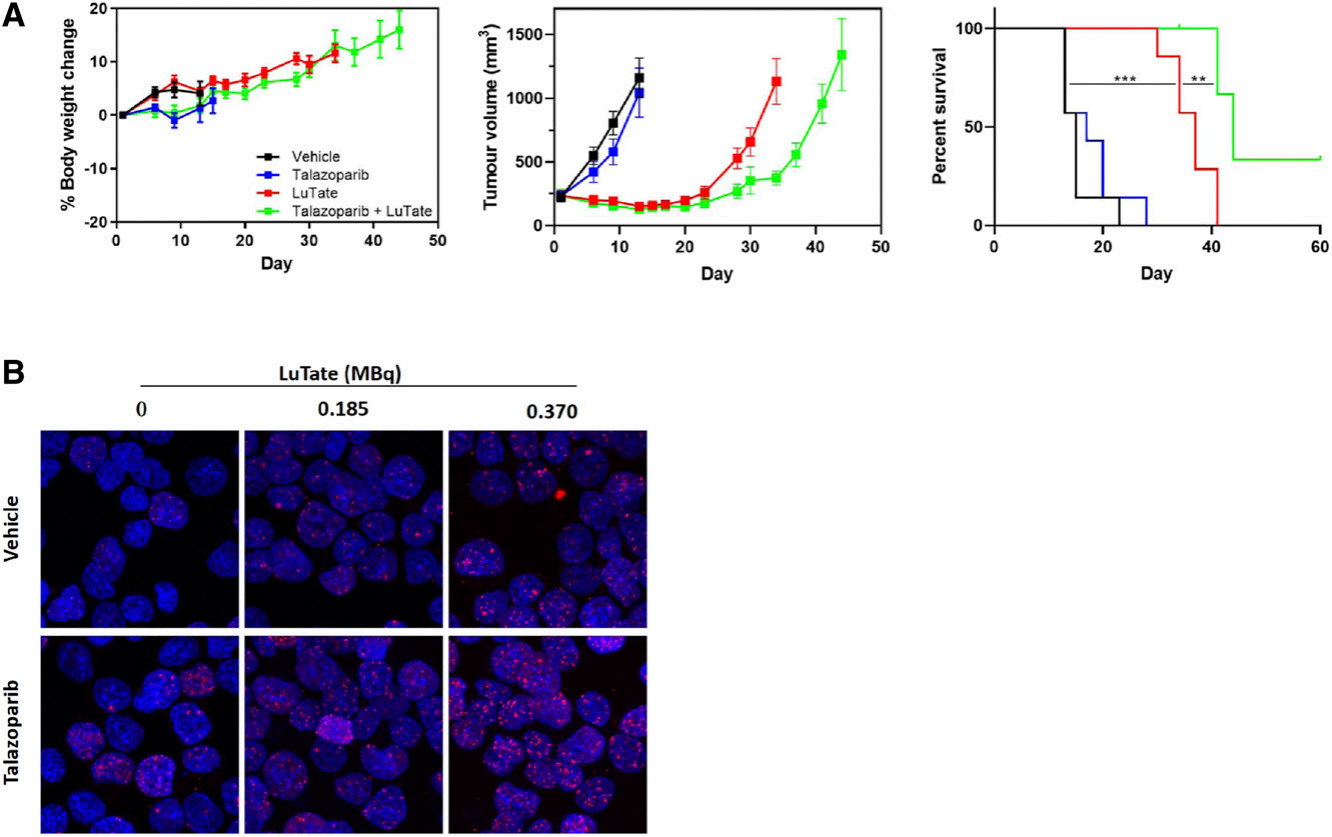
Evaluation of TRT + talazoparib treatment in exocrine pancreatic AR42J model. (A) Body weight, tumor volume, and percentage survival in AR42J tumor–bearing mice treated with 30 MBq of ^177^Lu-DOTATATE (day 1) with or without 0.25 mg/kg dose of talazoparib twice daily (days 1–5). (B) ɣH2AX staining of AR42J cells treated with ^177^Lu-DOTATATE with or without talazoparib for 2 h. (Reprinted with permission of (10).)

Although few studies have aimed to elucidate the mechanism behind PARP inhibitor–induced radiosensitization of TRT, ^177^Lu-DOTATATE + PARP inhibitor treatment has been associated with an increased number and persistence of double-stranded breaks; increased levels of DNA damage markers, such as γH2AX, phosphorylated p53, and 53BP1; and increased cell cycle arrest (Fig. 3) (8–10). Therefore, it has been proposed that PARP inhibition leads to an inability to repair the single-stranded breaks caused by ^177^Lu-DOTATATE, resulting in the formation of additional double-stranded breaks on cellular replication and, ultimately, cell death (8–10). Several clinical trials assessing the safety and efficacy of TRT + PARP inhibitors are under way (NCT04086485, NCT04375267, and NCT0387484), and the field is looking forward to their outcome. In the future, it would be of interest to compare the effects of TRT + PARP inhibitors in patients with deficient homologous recombination (e.g., with BRCA1/2 mutations) versus patients with functioning homologous recombination, as mutations in homologous recombination repair proteins are known to induce PARP inhibitor sensitivity (31).

In addition to PARP inhibitors, inhibitors of ataxia telangiectasia mutated, ataxia telangiectasia and Rad3-related, and DNA-dependent protein kinase, key effector proteins involved in DDR, have also been investigated as radiosensitizers. These combination studies are mainly with EBRT (32–34), but there are a few examples showing their ability to also radiosensitize TRT (35,36); however, it remains to be seen how these TRT combinations perform clinically. Combining TRT with inhibitors of epidermal growth factor receptor, which modulates DNA repair through activation of DNA-dependent protein kinase, has also been attempted in a few preclinical studies (37,38).

### HSP90 Inhibitors

^177^Lu-DOTATATE has been combined with the HSP90 inhibitors onalespib and ganetespib in vivo, with promising results and a favorable toxicity profile reported for NET xenografts (39,40). HSP90 is a molecular chaperone that participates in key processes such as protein degradation, folding, and intracellular transport. Clients for HSP90 include kinases involved in cell growth, such as epidermal growth factor receptor and protein kinase B, and DDR proteins, such as ataxia telangiectasia and Rad3-related and checkpoint kinase 1 (41,42). HSP90 inhibitors have been reported to induce radiosensitization when combined with EBRT, but because of the multiple clients associated with HSP90, the exact mechanism of HSP90 inhibitor–induced radiosensitization is unknown. However, it is known to negatively affect DNA repair signaling and the activation of cell cycle checkpoints, potentiating radiation-induced damage (43,44). To date, no study has sought to elucidate the mechanism of HSP90 inhibitor–induced radiosensitization in the context of TRT. Moreover, although several HSP90 inhibitors have been tested in clinical trials as potential monotherapies, a lack of efficacy and unacceptable toxicity have prevented any from being clinically approved, making this strategy challenging to translate to patients (45,46).

### Topoisomerase Inhibitors

Topoisomerases 1 and 2 are nuclear enzymes that are essential for maintaining the correct topological state of DNA, which is crucial for RNA transcription, chromatin remodelling, and DNA replication. Through the formation of a topoisomerase DNA covalent intermediate—the topoisomerase cleavable complex—the main role of topoisomerases 1 and 2 is to catalyze the relaxation of positive or negative DNA supercoiling by nicking DNA strands to allow controlled rotation, followed by religation (47). Many inhibitors of topoisomerases 1 and 2 work by stabilizing the topoisomerase cleavable complex, leading to the accumulation of single- and double-stranded breaks, respectively (47). In addition to creating DNA damage, numerous studies have shown that topoisomerase inhibitors can also act as radiosensitizers of EBRT (48–50); however, the precise radiosensitization mechanism remains unelucidated. Shih et al. suggested that topoisomerase inhibitor–mediated cytotoxicity and radiosensitization occur via different pathways, as the former is Ku80-independent whereas the latter is Ku80-dependent (51). Ku80 is required for nonhomologous end joining, suggesting that topoisomerase inhibitors can potentiate radiation-induced DNA damage through interfering with DDR. The schedule of topoisomerase inhibitor administration is also thought to be crucial for effective radiosensitization of EBRT, with varying degrees of radiosensitization observed depending on the dosing frequency (49,50).

Several studies investigated the use of topoisomerase 1 inhibitors with TRT, with the most frequently tested combination being ^131^I-metaiodobenzylguanidine + topotecan for the treatment of advanced neuroblastoma. In vitro and in vivo studies using the neuroblastoma SK-N-BE(2c) and glioma UVW/NAT models have shown that ^131^I-metaiodobenzylguanidine + topotecan treatment leads to superadditive DNA damage and reduced efficiency of DNA repair (52,53). As with EBRT + topoisomerase inhibitor combinations, the benefits of TRT + topoisomerase inhibitor treatment appears to depend on the timing of topoisomerase inhibitor administration: McCluskey et al. reported greater tumor growth delay in SK-N-BE(2C) and UVW/NAT tumor–bearing mice when topotecan was administered simultaneously with ^131^I-metaiodobenzylguanidine than when administered 24 h beforehand or afterward (52,53). This observation has been linked to differences in cell cycle distribution induced by the different schedules; however, further studies are needed for confirmation (53). Several clinical studies have been conducted with ^131^I-metaiodobenzylguanidine + topotecan in neuroblastoma patients, but it remains unclear whether the addition of topotecan leads to any significant benefit over ^131^I-metaiodobenzylguanidine monotherapy (Supplemental Table 2).

## RADIOSENSITIZATION THROUGH INHIBITING PHOSPHOINOSITIDE-3-KINASE/PROTEIN KINASE B/MAMMALIAN TARGET OF RAPAMYCIN (MTOR) SIGNALING

mTOR is a serine/threonine kinase that forms 2 complexes, mTOR complex 1 and mTOR complex 2, which differ in their subcellular location, structure, and function: mTOR complex 1 regulates cell growth and metabolism, and mTOR complex 2 regulates cell proliferation and survival (54). mTOR activity is regulated primarily by the phosphoinositide-3-kinase/protein kinase B/mTOR signaling pathway. In many human cancers, mTOR signaling is hyperactivated, leading to increased tumorigenesis, increased tumor progression, and decreased survival (55,56). mTOR is also known to influence DDR; for example, mTOR can positively and negatively regulate ataxia telangiectasia mutated (57).

Activation of the phosphoinositide-3-kinase/protein kinase B/mTOR pathway has been linked to the development of radioresistance, and multiple studies have investigated mTOR inhibitors for their potential radiosensitizing effects with EBRT (58–60). Multiple preclinical studies have demonstrated that EBRT + mTOR inhibitors leads to increased radiosensitivity through decreased expression of nonhomologous end joining and homologous recombination repair pathway proteins and increased expression of apoptosis pathway proteins (58–60). However, the use of mTOR inhibitors as radiosensitizers for TRT remains controversial, as preclinical studies investigating the efficacy of ^177^Lu-DOTATATE + mTOR inhibitors have reported contrasting results: Johnbeck et al. observed greater antitumor effects with ^177^Lu-DOTATATE + everolimus treatment than with ^177^Lu-DOTATATE alone in the mouse H727 non–small cell lung carcinoma model, Zellmer et al. found that the combination treatment was as effective as ^177^Lu-DOTATATE alone in the athymic mouse AR42J pancreatic model, and Pool et al. reported reduced antitumor effects with the combination treatment in the immunocompetent rat CA20948 pancreatic tumor model (61–64). Pool et al. also noted the development of distant metastases in more than 70% of the CA20948 rats in the everolimus-treated groups, whereas no metastases were observed in the control or ^177^Lu-DOTATATE–only groups (61,62). This increased propensity for metastasis formation was not observed in athymic AR42J tumor–bearing mice or in the phase I NETTLE trial evaluating the maximum tolerated dose of everolimus when administered with ^177^Lu-DOTATATE (64,65); thus, it is probable that the increased metastases observed are specific to the genotype of the CA20948 model. Alternatively, there is evidence that mTOR inhibition can influence endothelial–mesenchymal transition, with both the promotion and the suppression of endothelial–mesenchymal transition being observed when using mTOR inhibitors under different conditions (66,67). Endothelial–mesenchymal transition is known to contribute toward tumor progression and metastasis, and thus, the precise role of mTOR inhibitors on endothelial–mesenchymal transition when combined with TRT merits close investigation (68). Understanding the source of these increased metastases is crucial, particularly as a phase I/II trial aimed at assessing ^177^Lu-DOTATATE + everolimus treatment in NET patients is under way (NCT03629847).

In addition to its role in tumorigenesis, mTOR plays an essential part in immune system regulation—a fact that should be considered when designing TRT + mTOR inhibitor therapies. Everolimus is clinically used as an immunosuppressant because of its ability to promote the expansion of regulatory T cells (69). A study testing ^177^Lu-DOTATATE + everolimus in non–tumor-bearing rats saw no significant increases in renal or hematologic toxicities compared with ^177^Lu-DOTATATE treatment, but decreases in the white blood cell count were noted, in line with the immunosuppressive effects of everolimus (70). Although immunosuppression is typically undesirable in cancer treatment because it decreases immune surveillance, there is little evidence suggesting that using mTOR inhibitors as a monotherapy could promote tumor growth (71). Nevertheless, in LEW/SsNHsd Lewis rats—a substrain that shows an enhanced autoimmune response—bearing CA20948 tumors, Bison et al. observed complete tumor regression in 50% of the rats in the control group, compared with 12.5% in the group treated with everolimus alone (62). No cases of complete tumor regression were seen in control LEW/HanHsd rats, which possess a less active immune response (62). Both findings indicate immune system involvement and suggest that any immunosuppressive effects should be considered when developing treatment combinations with TRT.

## RADIOSENSITIZATION THROUGH INHIBITING HEDGEHOG SIGNALING

The Hedgehog signaling pathway is involved in embryonic development and is abnormally activated in many cancers. Inhibitors of the transmembrane protein smoothened, a key component of the Hedgehog pathway, have been investigated as potential anticancer drugs, with several of these inhibitors now approved by the U.S. Food and Drug Administration. Spetz et al. showed that combining ^177^Lu-DOTATATE with the smoothened antagonist sonidegib in GOT1 tumor–bearing mice led to an increased time to progression compared with the respective monotherapies (72). Moreover, pathway analysis predicted that this combination therapy impacted cancer related pathways, such as Wnt/β-catenin, Notch, and nuclear factor κ-light-chain enhancer, differently from either sonidegib or ^177^Lu-DOTATATE alone (72). Although experimental validation is required for these predictions, this finding suggests that radiosensitization of ^177^Lu-DOTATATE by sonidegib could involve multiple biological pathways and may therefore be challenging to optimize.

## RADIOSENSITIZATION THROUGH INHIBITING P53–MURINE DOUBLE MINUTE 2 (MDM2) INTERACTIONS

The tumor suppressor protein p53 regulates cell cycle progression, DNA repair, and apoptosis and is negatively regulated by MDM2. MDM2 is overexpressed in many human tumors, leading to decreased p53 activity; thus, disruption of the p53–MDM2 interaction is a promising therapeutic strategy. Several studies have shown that ionizing radiation induces p53-dependent MDM2 expression, and p53/MDM2 inhibitors may therefore act as radiosensitizers by promoting p53-dependent apoptosis (73,74). Despite the fact that p53–MDM2 inhibitors are being investigated as radiosensitizers for EBRT in multiple studies, there are few examples of them being used with TRT to date and further studies are needed to validate this approach (Supplemental Table 5). However, this strategy depends on the presence of wild-type p53 and may therefore not be as effective in cancers in which *TP53* mutations are common, such as metastatic castration-resistant prostate cancer (75,76).

## RADIOSENSITIZATION THROUGH DISRUPTING CELL CYCLE

Microtubules, composed of heterodimers of α- and β-tubulin, are essential for cell signaling, division, and mitosis and for maintaining cellular structure (77). Taxane drugs stabilize microtubules by binding to β-tubulin, promoting microtubule polymerization and, ultimately, G2/M cell cycle arrest and apoptosis (77). The cell cycle plays an important role in radiosensitivity, with cells being most sensitive in the G2/M phases and most resistant in the S phase (78). Because of their effect on the cell cycle, there are many examples of the use of taxanes as radiosensitizers for EBRT, with encouraging outcomes observed (79). However, only a few TRT + taxane combination therapies have been investigated; although promising preclinical results have been reported for ^177^Lu- and ^188^Re-based TRTs, Kessel et al. reported that the benefit of ^177^Lu-PSMA was decreased in patients with a history of second-line cabazitaxel therapy, suggesting that further investigation into the mechanism of TRT + taxane combinations is warranted (Supplemental Table 6) (80). In particular, the sequencing of the therapies is likely to be important, as ionizing radiation itself influences the cell cycle (81); Liebmann et al. noted that irradiating human breast MCF-7 and lung A549 adenocarcinoma cells with EBRT before or concurrently with paclitaxel antagonized paclitaxel cytotoxicity (82). Optimizing this sequencing with TRT is likely to be challenging as, in contrast to EBRT, TRT delivers radiation heterogeneously over an extended period with less temporal control over the absorbed radiation dose (1).

## RADIOSENSITIZATION THROUGH DISRUPTING NICOTINAMIDE ADENINE DINUCLEOTIDE (NAD+) METABOLISM

Nicotinamide phosphoribosyl transferase (NAMPT) is an enzyme that is essential for NAD+ metabolism. NAD+ is required for many cellular processes, including the activation of PARP-1, and is regenerated via a salvage pathway involving NAMPT. NAMPT inhibitors have been proposed to work as radiosensitizers of TRT by preventing NAD+ regeneration; on PARP-1 activation due to TRT-induced DNA damage, NAD+ is consumed and cannot be regenerated, leading it to drop to lethally low levels (83,84). To date, there has been just one investigation of TRT + NAMPT inhibitors: Elf et al. showed that the combination of ^177^Lu-DOTATATE and the experimental NAMPT inhibitor GMX1778 led to reduced tumor volumes and prolonged antitumor response in GOT1 tumor–bearing mice (83). However, more studies are required to assess the efficacy of this strategy and determine the exact radiosensitization mechanism. Moreover, since NAD+ is involved in many other processes, this approach could lead to off-target effects.

## RADIOSENSITIZATION THROUGH BLOCKING IMMUNE CHECKPOINTS

Irradiation in the form of EBRT is known to have several immunomodulatory effects; for instance, EBRT can enhance tumor immunogenicity by inducing immunogenic cell death and promoting the release of tumor-associated antigens while simultaneously reducing tumor immunogenicity by upregulating programmed death ligand 1 expression (85). Combining EBRT with immune checkpoint blockade antibodies targeting programmed death 1, programmed death ligand 1, and cytotoxic T-lymphocyte antigen 4 represents an attractive strategy to potentiate radiation-induced antitumor immunity and has produced encouraging results (86,87). Although little is currently known about the effects of TRT on tumor immunogenicity, preclinical studies have shown that TRT also leads to upregulation of programmed death ligand 1 expression and that combinations of TRT with immune checkpoint blockade can lead to improved survival (11–13). Several clinical trials combining TRT + programmed death 1 inhibitors are currently under way (e.g., NCT03325816 and NCT03658447), though it is likely that further optimization of when immunotherapy should be administered during TRT treatment will be required; as with EBRT, Chen et al. noted in their preclinical studies that the benefits of combining TRT + anti-programmed death ligand 1 varies depending on whether the two treatments are given concurrently or sequentially (12,86).

## OUTLOOK

The rising interest in TRT has led to the evaluation of numerous combination strategies, with promising results being reported and increasing numbers of clinical trials being conducted. In addition to the combination strategies discussed here, alternative strategies that have been proposed include those aimed at increasing the cellular uptake of radiopharmaceuticals and the rates of tumor perfusion (3,4). Multiple studies have also investigated the combination of TRT with EBRT, as well as with other β- or α-emitting TRTs (88–90). Growing numbers of triple-combination therapies are also being evaluated, such as TRT + CAPTEM (NCT02358356) and TRT + vincristine + irinotecan (NCT01313936), though it remains to be seen whether these strategies are effective in increasing the therapeutic index.

Despite the growing number of studies being conducted in this field, there remain few key outcomes. Increasing our understanding of TRT radiobiology is critical if we are to fully capitalize on the potential benefits of these combination therapies. For instance, the synergistic effects of taxanes and topoisomerase inhibitors in combination with TRT are believed to be cell cycle–dependent, and the effects of both the drugs and TRT on the cell cycle must be understood before we can optimize the dosing schedule (53,81,82). Greater characterization of the tested combinations over a range of concentrations is also necessary to identify combinations that are superadditive, as opposed to simply additive or even antagonistic. Moreover, many of the current combination strategies being used for TRT involve known radiosensitizers for EBRT, despite its markedly different radiobiology (1,15,16). Although these strategies may identify combinations that are effective with both forms of ionizing radiation, it is likely that there exist effective radiosensitizers for TRT that are ineffective when combined with EBRT and vice versa. In addition, although this review focused on β-emitting TRTs, there is growing interest in the use of α-and Auger emitters (91–93). However, because of differences in radiobiology and therefore biologically effective dose, effective radiosensitizers of β-emitting TRTs may not be effective radiosensitizers of α- and Auger-emitting TRTs. Differences may also exist between different β-emitters due to differences in dose rates and dose deposition profiles.

Furthermore, many of the commonly used preclinical models thus far are not entirely clinically relevant; for example, despite the use of U2OS cells expressing somatostatin receptor 2 and BON-1 cells to evaluate ^177^Lu-DOTATATE combination therapies, U2OS cells expressing somatostatin receptor 2 is a non-NET cell line and BON-1 cells express much lower levels of somatostatin receptor than are found in human NET tumors (4,8). BON-1 cells also show mutations of key DDR genes such as *TP53*—mutations that are rarely seen in G1 and G2 NETs (94,95). Preclinical studies on a greater variety of models are necessary, as well as identification of more clinically relevant model systems. Most preclinical studies to date have been on immune-compromised mice, and possible influences of the immune system on combination therapies and vice versa have not been explored in detail.

Moving forward, an increasing mechanistic understanding of TRT-based combination therapies is crucial if these strategies are to be effectively—and safely—translated to patients, particularly given the outcome of the phase III ERA223 trial, in which abiraterone acetate + prednisone/prednisolone + ^223^Ra resulted in no improvement in survival compared with abiraterone acetate + prednisone/prednisolone alone but was associated with an increased frequency of skeletal fractures (96). Similarly, initial results from the phase I/II CAPTEM trial (NCT02358356) show that although ^177^Lu-DOTATATE + CAPTEM treatment led to higher objective tumor-response rates than ^177^Lu-DOTATATE alone, the former was associated with more treatment-related adverse effects (20). Recent evidence has also alluded to better survival for chemotherapy-naïve patients treated with TRT than for those with a prior history of chemotherapy, possibly due to the acquisition of resistance mechanisms as has been suggested for second and subsequent lines of chemotherapy (25,97). The use of TRT combination therapies as first-line treatments may therefore produce greater survival benefits and should be explored further. In addition to potentially increasing therapeutic efficacy and minimizing the occurrence of toxicities, greater understanding of the radiobiology behind these combination strategies may also allow us to stratify patients and tailor combination therapies to the grade and mutational landscape of each patient’s cancer.

## DISCLOSURE

Funding was provided to Tiffany Chan by Prostate Cancer Research Centre (U.K.), to Edward O’Neill by UKRI/MRC, and to Bart Cornelissen by UKRI/MRC and Cancer Research U.K. through the Oxford Institute for Radiation Biology. Bart Cornelissen serves as a consultant for Theragnostics Ltd. No other potential conflict of interest relevant to this article was reported.

## References

[1] Pouget J-P Lozza C Deshayes E Boudousq V Navarro-Teulon I. Introduction to radiobiology of targeted radionuclide therapy. Front Med (Lausanne). 2015;2:12. 25853132 10.3389/fmed.2015.00012 PMC4362338

[2] Strosberg J El-Haddad G Wolin E. Phase 3 trial of ^177^Lu-DOTATATE for midgut neuroendocrine tumors. N Engl J Med. 2017;376:125–135. 28076709 10.1056/NEJMoa1607427 PMC5895095

[3] Bison SM Haeck JC Bol K. Optimization of combined temozolomide and peptide receptor radionuclide therapy (PRRT) in mice after multimodality molecular imaging studies. EJNMMI Res. 2015;5:62. 26553049 10.1186/s13550-015-0142-y PMC4639542

[4] Taelman VF Radojewski P Marincek N. Upregulation of key molecules for targeted imaging and therapy. J Nucl Med. 2016;57:1805–1810. 27363833 10.2967/jnumed.115.165092

[5] Claringbold PG Brayshaw PA Price RA Turner JH. Phase II study of radiopeptide ^177^Lu-octreotate and capecitabine therapy of progressive disseminated neuroendocrine tumours. Eur J Nucl Med Mol Imaging. 2011;38:302–311. 21052661 10.1007/s00259-010-1631-x

[6] Yordanova A Ahrens H Feldmann G. Peptide receptor radionuclide therapy combined with chemotherapy in patients with neuroendocrine tumors. Clin Nucl Med. 2019;44:e329–e335. 30932975 10.1097/RLU.0000000000002532

[7] Claringbold PG Price RA Turner JH. Phase I-II study of radiopeptide ^177^Lu-octreotate in combination with capecitabine and temozolomide in advanced low-grade neuroendocrine tumors. Cancer Biother Radiopharm. 2012;27:561–569. 23078020 10.1089/cbr.2012.1276

[8] Purohit NK Shah RG Adant S Hoepfner M Shah GM Beauregard JM. Potentiation of ^177^Lu-octreotate peptide receptor radionuclide therapy of human neuroendocrine tumor cells by PARP inhibitor. Oncotarget. 2018;9:24693–24706. 29872498 10.18632/oncotarget.25266 PMC5973847

[9] Nonnekens J van Kranenburg M Beerens CEMT. Potentiation of peptide receptor radionuclide therapy by the PARP inhibitor olaparib. Theranostics. 2016;6:1821–1832. 27570553 10.7150/thno.15311 PMC4997239

[10] Cullinane C Waldeck K Kirby L. Enhancing the anti-tumour activity of ^177^Lu-DOTA-octreotate radionuclide therapy in somatostatin receptor-2 expressing tumour models by targeting PARP. Sci Rep. 2020;10:10196. 32576907 10.1038/s41598-020-67199-9 PMC7311440

[11] Czernin J Current K Mona CE. Immune-checkpoint blockade enhances ^225^Ac-PSMA617 efficacy in a mouse model of prostate cancer. J Nucl Med. July 9, 2020 [Epub ahead of print]. 10.2967/jnumed.120.246041 PMC12079179 32646877

[12] Chen H Zhao L Fu K. Integrin αvβ3-targeted radionuclide therapy combined with immune checkpoint blockade immunotherapy synergistically enhances anti-tumor efficacy. Theranostics. 2019;9:7948–7960. 31695808 10.7150/thno.39203 PMC6831469

[13] Choi J Beaino W Fecek RJ. Combined VLA-4–targeted radionuclide therapy and immunotherapy in a mouse model of melanoma. J Nucl Med. 2018;59:1843–1849. 29959213 10.2967/jnumed.118.209510 PMC6278902

[14] Kletting P Thieme A Eberhardt N. Modeling and predicting tumor response in radioligand therapy. J Nucl Med. 2019;60:65–70. 29748236 10.2967/jnumed.118.210377

[15] O’Donoghue J. Relevance of external beam dose-response relationships to kidney toxicity associated with radionuclide therapy. Cancer Biother Radiopharm. 2004;19:378–387. 15285886 10.1089/1084978041425025

[16] Terry SYA Nonnekens J Aerts A. Call to arms: need for radiobiology in molecular radionuclide therapy. Eur J Nucl Med Mol Imaging. 2019;46:1588–1590. 31069454 10.1007/s00259-019-04334-3

[17] Feijtel D de Jong M Nonnekens J. Peptide receptor radionuclide therapy: looking back, looking forward. Curr Top Med Chem. February 25, 2020 [Epub ahead of print]. 10.2174/1568026620666200226104652 PMC8493789 32101125

[18] Ballal S Yadav MP Damle NA Sahoo RK Bal C. Concomitant ^177^Lu-DOTATATE and capecitabine therapy in patients with advanced neuroendocrine tumors: a long-term-outcome, toxicity, survival, and quality-of-life study. Clin Nucl Med. 2017;42:e457–e466. 28872545 10.1097/RLU.0000000000001816

[19] van Essen M Krenning EP Kam BL De Herder WW Van Aken MO Kwekkeboom DJ. Report on short-term side effects of treatments with ^177^Lu-octreotate in combination with capecitabine in seven patients with gastroenteropancreatic neuroendocrine tumours. Eur J Nucl Med Mol Imaging. 2008;35:743–748. 18188559 10.1007/s00259-007-0688-7 PMC2668587

[20] Pavlakis N Ransom DT Wyld D. First results for Australasian Gastrointestinal Trials Group (AGITG) control net study: phase II study of ^177^Lu-octreotate peptide receptor radionuclide therapy (LuTate PRRT) +/− capecitabine, temozolomide (CAPTEM) for midgut neuroendocrine tumors (mNETs) [abstract]. J Clin Oncol. 2020;38(suppl):604.

[21] Chatzellis E Angelousi A Daskalakis K. Activity and safety of standard and prolonged capecitabine/temozolomide administration in patients with advanced neuroendocrine neoplasms. Neuroendocrinology. 2019;109:333–345. 31167197 10.1159/000500135

[22] Fine RL Gulati AP Krantz BA. Capecitabine and temozolomide (CAPTEM) for metastatic, well-differentiated neuroendocrine cancers: the Pancreas Center at Columbia University experience. Cancer Chemother Pharmacol. 2013;71:663–670. 23370660 10.1007/s00280-012-2055-z

[23] Ahmadzadehfar H Rahbar K Baum RP. Prior therapies as prognostic factors of overall survival in metastatic castration-resistant prostate cancer patients treated with [^177^Lu]Lu-PSMA-617: a WARMTH multicenter study (the 617 trial). Eur J Nucl Med Mol Imaging. May 8, 2020 [Epub ahead of print]. 10.1007/s00259-020-04797-9 PMC7835179 32383093

[24] Kulkarni H Schuchardt C, Singh A, Langbein T, Baum R. Early initiation of Lu-177 PSMA radioligand therapy prolongs overall survival in metastatic prostate cancer [abstract]. J Nucl Med. 2018;59(suppl):529.29025984

[25] Sathekge M Bruchertseifer F Knoesen O. ^225^Ac-PSMA-617 in chemotherapy-naive patients with advanced prostate cancer: a pilot study. Eur J Nucl Med Mol Imaging. 2019;46:129–138. 30232539 10.1007/s00259-018-4167-0 PMC6267694

[26] Zhou JX, Feng LJ, Zhang X. Risk of severe hematologic toxicities in cancer patients treated with PARP inhibitors: a meta-analysis of randomized controlled trials. Drug Des Devel Ther. 2017;11:3009–3017. 10.2147/DDDT.S147726 PMC5648323 29075104

[27] Boles NC Peddibhotla S Chen AJ Goodell MA Rosen JM. Chk1 haploinsufficiency results in anemia and defective erythropoiesis. *PLoS One*. 2010;5:e8581. 20052416 10.1371/journal.pone.0008581 PMC2798715

[28] Ray Chaudhuri A Nussenzweig A. The multifaceted roles of PARP1 in DNA repair and chromatin remodelling. Nat Rev Mol Cell Biol. 2017;18:610–621. 28676700 10.1038/nrm.2017.53 PMC6591728

[29] Camero S Ceccarelli S De Felice F. PARP inhibitors affect growth, survival and radiation susceptibility of human alveolar and embryonal rhabdomyosarcoma cell lines. J Cancer Res Clin Oncol. 2019;145:137–152. 30357520 10.1007/s00432-018-2774-6 PMC6326011

[30] Lesueur P Chevalier F Austry JB. Poly-(ADP-ribose)-polymerase inhibitors as radiosensitizers: a systematic review of pre-clinical and clinical human studies. Oncotarget. 2017;8:69105–69124. 28978184 10.18632/oncotarget.19079 PMC5620324

[31] Farmer H McCabe H Lord CJ. Targeting the DNA repair defect in BRCA mutant cells as a therapeutic strategy. Nature. 2005;434:917–921. 15829967 10.1038/nature03445

[32] Ismail IH Mårtensson S Moshinsky D. SU11752 inhibits the DNA-dependent protein kinase and DNA double-strand break repair resulting in ionizing radiation sensitization. Oncogene. 2004;23:873–882. 14661061 10.1038/sj.onc.1207303

[33] Rainey MD Charlton ME Stanton RV Kastan MB. Transient inhibition of ATM kinase is sufficient to enhance cellular sensitivity to ionizing radiation. Cancer Res. 2008;68:7466–7474. 18794134 10.1158/0008-5472.CAN-08-0763 PMC2559948

[34] Wengner AM Siemeister G Lucking U. The novel ATR inhibitor BAY 1895344 is efficacious as monotherapy and combined with DNA damage–inducing or repair–compromising therapies in preclinical cancer models. Mol Cancer Ther. 2020;19:26–38. 31582533 10.1158/1535-7163.MCT-19-0019

[35] Wickstroem K Hagemann UB Cruciani V. Synergistic effect of a mesothelin-targeted ^227^Th conjugate in combination with DNA damage response inhibitors in ovarian cancer xenograft models. J Nucl Med. 2019;60:1293–1300. 30850485 10.2967/jnumed.118.223701 PMC6735281

[36] Song H Hedayati M Hobbs RF. Targeting aberrant DNA double-strand break repair in triple-negative breast cancer with alpha-particle emitter radiolabeled anti-EGFR antibody. Mol Cancer Ther. 2013;12:2043–2054. 23873849 10.1158/1535-7163.MCT-13-0108 PMC3804319

[37] Vassileva V Rajkumar V Mazzantini M. Significant therapeutic efficacy with combined radioimmunotherapy and cetuximab in preclinical models of colorectal cancer. J Nucl Med. 2015;56:1239–1245. 26045312 10.2967/jnumed.115.157362

[38] Kelly MP Lee ST Lee FT. Therapeutic efficacy of ^177^Lu-CHX-A″-DTPA-hu3SI93 radioimmunotherapy in prostate cancer is enhanced by EGFR inhibition or docetaxel chemotherapy. Prostate. 2009;69:92–104. 18942092 10.1002/pros.20856 PMC2597150

[39] Hofving T Sandblom V Arvidsson Y. ^177^Lu-octreotate therapy for neuroendocrine tumours is enhanced by Hsp90 inhibition. Endocr Relat Cancer. 2019;26:437–449. 30730850 10.1530/ERC-18-0509 PMC6391910

[40] Lundsten S Spiegelberg D Raval NR Nestor M. The radiosensitizer onalespib increases complete remission in ^177^Lu-DOTATATE-treated mice bearing neuroendocrine tumor xenografts. Eur J Nucl Med Mol Imaging. 2020;47:980–990. 31912256 10.1007/s00259-019-04673-1 PMC7075859

[41] Basso AD Solit DB Chiosis G Giri B Tsichlis P Rosen N. Akt forms an intracellular complex with heat shock protein 90 (Hsp90) and Cdc37 and is destabilized by inhibitors of Hsp90 function. J Biol Chem. 2002;277:39858–39866. 12176997 10.1074/jbc.M206322200

[42] Pennisi R Ascenzi P di Masi A. Hsp90: a new player in DNA repair? Biomolecules. 2015;5:2589–2618. 26501335 10.3390/biom5042589 PMC4693249

[43] Lee Y Sunada S Hirakawa H Fujimori A Nickoloff JA Okayasu R. TAS-116, a novel hsp90 inhibitor, selectively enhances radiosensitivity of human cancer cells to x-rays and carbon ion radiation. Mol Cancer Ther. 2017;16:16–24. 28062703 10.1158/1535-7163.MCT-16-0573 PMC5221699

[44] Dote H Burgan WE Camphausen K Tofilon PJ. Inhibition of Hsp90 compromises the DNA damage response to radiation. Cancer Res. 2006;66:9211–9220. 16982765 10.1158/0008-5472.CAN-06-2181

[45] Johnson ML Yu HA Hart EM. Phase I/II study of HSP90 inhibitor AUY922 and erlotinib for EGFR-mutant lung cancer with acquired resistance to epidermal growth factor receptor tyrosine kinase inhibitors. J Clin Oncol. 2015;33:1666–1673. 25870087 10.1200/JCO.2014.59.7328 PMC4881377

[46] Walker AR Klisovic R Johnston JS. Pharmacokinetics and dose escalation of the heat shock protein inhibitor 17-allyamino-17-demethoxygeldanamycin in combination with bortezomib in relapsed or refractory acute myeloid leukemia. Leuk Lymphoma. 2013;54:1996–2002. 23256542 10.3109/10428194.2012.760733 PMC3860322

[47] Pommier Y. Topoisomerase I inhibitors: camptothecins and beyond. Nat Rev Cancer. 2006;6:789–802. 16990856 10.1038/nrc1977

[48] Chen AY Okunieff P Pommier Y Mitchell JB. Mammalian DNA topoisomerase I mediates the enhancement of radiation cytotoxicity by camptothecin derivatives. Cancer Res. 1997;57:1529–1536. 9108456

[49] Kirichenko AV Rich TA Newman RA Travis EL. Potentiation of murine MCA-4 carcinoma radioresponse by 9-amino-20(S)-camptothecin. Cancer Res. 1997;57:1929–1933. 9157987

[50] Kim JH Kim SH Kolozsvary A Khil MS. Potentiation of radiation response in human carcinoma cells in vitro and murine fibrosarcoma in vivo by topotecan, an inhibitor of DNA topoisomerase I. Int J Radiat Oncol Biol Phys. 1992;22:515–518. 1310495 10.1016/0360-3016(92)90865-f

[51] Shih SJ Erbele T Chen AY. Ku86 modulates DNA topoisomerase I-mediated radiosensitization, but not cytotoxicity, in mammalian cells. Cancer Res. 2005;65:9194–9199. 16230379 10.1158/0008-5472.CAN-05-2387

[52] McCluskey AG Boyd M Ross SC. [^131^I]meta-iodobenzylguanidine and topotecan combination treatment of tumors expressing the noradrenaline transporter. Clin Cancer Res. 2005;11:7929–7937. 16278418 10.1158/1078-0432.CCR-05-0982

[53] McCluskey AG Boyd M Pimlott SL Babich JW Gaze MN Mairs RJ. Experimental treatment of neuroblastoma using [^131^I]meta-iodobenzylguanidine and topotecan in combination. Br J Radiol. 2008;81(suppl):S28–S35. 18819996 10.1259/bjr/27723093

[54] Saxton RA Sabatini DM. mTOR signaling in growth, metabolism, and disease. Cell. 2017;168:960–976. 28283069 10.1016/j.cell.2017.02.004 PMC5394987

[55] Laes JF Sauvage S Ghitti G. Tumor-biopsy stratification based on mTOR-pathway activity and functional mutations in the upstream genes PIK3CA and PTEN. Oncotarget. 2017;8:84426–84433. 29137436 10.18632/oncotarget.21348 PMC5663608

[56] Leal P Garćia P Sandoval A. Immunohistochemical expression of phospho-mTOR is associated with poor prognosis in patients with gallbladder adenocarcinoma. Arch Pathol Lab Med. 2013;137:552–557. 23544944 10.5858/arpa.2012-0032-OA

[57] Ma Y Vassetzky Y Dokudovskaya S. mTORC1 pathway in DNA damage response. Biochim Biophys Acta Mol Cell Res. 2018;1865:1293–1311. 29936127 10.1016/j.bbamcr.2018.06.011

[58] Yu C-C Hung S-K Lin H-Y. Targeting the PI3K/AKT/mTOR signaling pathway as an effectively radiosensitizing strategy for treating human oral squamous cell carcinoma in vitro and in vivo. Oncotarget. 2017;8:68641–68653. 28978144 10.18632/oncotarget.19817 PMC5620284

[59] Chen H Ma Z Vanderwaal RP. The mTOR inhibitor rapamycin suppresses DNA double-strand break repair. Radiat Res. 2011;175:214–224. 21268715 10.1667/rr2323.1 PMC4412148

[60] Chang L Graham PH Hao J. PI3K/Akt/mTOR pathway inhibitors enhance radiosensitivity in radioresistant prostate cancer cells through inducing apoptosis, reducing autophagy, suppressing NHEJ and HR repair pathways. Cell Death Dis. 2014;5:e1437. 25275598 10.1038/cddis.2014.415 PMC4237243

[61] Pool SE Bison S Koelewijn SJ. mTOR inhibitor RAD001 promotes metastasis in a rat model of pancreatic neuroendocrine cancer. Cancer Res. 2013;73:12–18. 23149918 10.1158/0008-5472.CAN-11-2089

[62] Bison SM Pool SE Koelewijn SJ. Peptide receptor radionuclide therapy (PRRT) with [^177^Lu-DOTA^0^,Tyr^3^]octreotate in combination with RAD001 treatment: further investigations on tumor metastasis and response in the rat pancreatic CA20948 tumor model. EJNMMI Res. 2014;4:21. 24995150 10.1186/s13550-014-0021-y PMC4070081

[63] Johnbeck CB Nielsen C Knigge U Kjaer A. Synergistic effect of combined treatment with ^177^Lu-DOTATATE and everolimus in neuroendocrine tumors as monitored by ^18^F-FDG-PET: studies in human neuroendocrine xenografts [abstract]. J Nucl Med. 2012;53(suppl):57.

[64] Zellmer J Vomacka L Boening G. Combination of peptide receptor radionuclide therapy with Lu-177 DOTATATE and the m-TOR inhibitor RAD001 (everolimus) in AR42J tumor bearing mice and response assessment by Ga-68 DOTATATE PET [abstract]. J Nucl Med. 2018;59(suppl):1346b.

[65] Claringbold PG Turner JH. NeuroEndocrine tumor therapy with lutetium-177-octreotate and everolimus (NETTLE): a phase I study. Cancer Biother Radiopharm. 2015;30:261–269. 26181854 10.1089/cbr.2015.1876

[66] Sasaki N Itakura Y Toyoda M. Rapamycin promotes endothelial-mesenchymal transition during stress-induced premature senescence through the activation of autophagy. Cell Commun Signal. 2020;18:43. 32164764 10.1186/s12964-020-00533-w PMC7069020

[67] Tian D Zeng X Wang W Wang Z Zhang Y Wang Y. Protective effect of rapamycin on endothelial-to-mesenchymal transition in HUVECs through the Notch signaling pathway. Vascul Pharmacol. 2019;113:20–26. 30336218 10.1016/j.vph.2018.10.004

[68] Potenta S Zeisberg E Kalluri R. The role of endothelial-to-mesenchymal transition in cancer progression. Br J Cancer. 2008;99:1375–1379. 18797460 10.1038/sj.bjc.6604662 PMC2579683

[69] Huijts CM Santegoets SJ de Jong TD Verheul HM de Gruijl TD van der Vliet HJ. Immunological effects of everolimus in patients with metastatic renal cell cancer. Int J Immunopathol Pharmacol. 2017;30:341–352. 28988508 10.1177/0394632017734459 PMC5806813

[70] Zellmer J Yen H-Y Kaiser L. Toxicity of a combined therapy using the mTOR-inhibitor everolimus and PRRT with [^177^Lu]Lu-DOTA-TATE in Lewis rats. EJNMMI Res. 2020;10:41. 32335736 10.1186/s13550-020-00628-y PMC7183514

[71] Law BK. Rapamycin: an anti-cancer immunosuppressant? Crit Rev Oncol Hematol. 2005;56:47–60. 16039868 10.1016/j.critrevonc.2004.09.009

[72] Spetz J Langen B Rudqvist N. Hedgehog inhibitor sonidegib potentiates ^177^Lu-octreotate therapy of GOT1 human small intestine neuroendocrine tumors in nude mice. BMC Cancer. 2017;17:528. 28789624 10.1186/s12885-017-3524-x PMC5549301

[73] Arya AK El-Fert A Devling T. Nutlin-3, the small-molecule inhibitor of MDM2, promotes senescence and radiosensitises laryngeal carcinoma cells harbouring wild-type p53. Br J Cancer. 2010;103:186–195. 20588277 10.1038/sj.bjc.6605739 PMC2906734

[74] Cao C Shinohara ET Subhawong TK. Radiosensitization of lung cancer by nutlin, an inhibitor of murine double minute 2. Mol Cancer Ther. 2006;5:411–417. 16505116 10.1158/1535-7163.MCT-05-0356

[75] Konukiewitz B Schlitter AM Jesinghaus M. Somatostatin receptor expression related to TP53 and RB1 alterations in pancreatic and extrapancreatic neuroendocrine neoplasms with a Ki67-index above 20%. Mod Pathol. 2017;30:587–598. 28059098 10.1038/modpathol.2016.217

[76] Robinson D Van Allen EM Wu YM. Integrative clinical genomics of advanced prostate cancer. Cell. 2015;161:1215–1228. 26000489 10.1016/j.cell.2015.05.001 PMC4484602

[77] McGrogan BT Gilmartin B Carney DN McCann A. Taxanes, microtubules and chemoresistant breast cancer. Biochim Biophys Acta. 2008;1785:96–132. 18068131 10.1016/j.bbcan.2007.10.004

[78] Sinclair WK. Cyclic x-ray responses in mammalian cells in vitro. Radiat Res. 1968;33:620–643. 4867897

[79] Golden EB Formenti SC Schiff PB. Taxanes as radiosensitizers. Anticancer Drugs. 2014;25:502–511. 24335716 10.1097/CAD.0000000000000055

[80] Kessel K Seifert R Schäfers M. Second line chemotherapy and visceral metastases are associated with poor survival in patients with mCRPC receiving ^177^Lu-PSMA-617. Theranostics. 2019;9:4841–4848. 31410185 10.7150/thno.35759 PMC6691377

[81] Bernhard EJ Maity A Muschel RJ McKenna WG. Effects of ionizing radiation on cell cycle progression: a review. Radiat Environ Biophys. 1995;34:79–83. 7652155 10.1007/BF01275210

[82] Liebmann J. Antagonism of paclitaxel cytotoxicity by x-rays: implications for the sequence of combined modality therapy. Int J Oncol. 1996;8:991–996. 21544456 10.3892/ijo.8.5.991

[83] Elf AK Bernhardt P Hofving T. NAMPT inhibitor GMX1778 enhances the efficacy of ^177^Lu-DOTATATE treatment of neuroendocrine tumors. J Nucl Med. 2017;58:288–292. 27688470 10.2967/jnumed.116.177584

[84] Kato H Ito E Shi W. Efficacy of combining GMX1777 with radiation therapy for human head and neck carcinoma. Clin Cancer Res. 2010;16:898–911. 20103674 10.1158/1078-0432.CCR-09-1945

[85] Pitroda SP Chmura SJ Weichselbaum RR. Integration of radiotherapy and immunotherapy for treatment of oligometastases. Lancet Oncol. 2019;20:e434–e442. 31364595 10.1016/S1470-2045(19)30157-3

[86] Pomeranz Krummel DA Nasti TH Izar B. Impact of sequencing radiation therapy and immune checkpoint inhibitors in the treatment of melanoma brain metastases. Int J Radiat Oncol Biol Phys. 2020;108:157–163. 32057994 10.1016/j.ijrobp.2020.01.043 PMC7839060

[87] Twyman-Saint Victor C Rech AJ Maity A. Radiation and dual checkpoint blockade activate non-redundant immune mechanisms in cancer. Nature. 2015;520:373–377. 25754329 10.1038/nature14292 PMC4401634

[88] Corroyer-Dulmont A Falzone N Kersemans V. Improved outcome of ^131^I-mIBG treatment through combination with external beam radiotherapy in the SK-N-SH mouse model of neuroblastoma. Radiother Oncol. 2017;124:488–495. 28595752 10.1016/j.radonc.2017.05.002 PMC5636618

[89] de Jong M Breeman WAP Valkema R Bernard BF Krenning EP. Combination radionuclide therapy using ^177^Lu and ^90^Y-labeled somatostatin analogs. J Nucl Med. 2005;46(suppl):13S–17S. 15653647

[90] Khreish F Ebert N Ries M. ^225^Ac-PSMA-617/^177^Lu-PSMA-617 tandem therapy of metastatic castration-resistant prostate cancer: pilot experience. Eur J Nucl Med Mol Imaging. 2020;47:721–728. 31758224 10.1007/s00259-019-04612-0

[91] Kratochwil C Haberkorn U Giesel FL. ^225^Ac-PSMA-617 for therapy of prostate cancer. Semin Nucl Med. 2020;50:133–140. 32172798 10.1053/j.semnuclmed.2020.02.004

[92] Navalkissoor S Grossman A. Targeted alpha particle therapy for neuroendocrine tumours: the next generation of peptide receptor radionuclide therapy. Neuroendocrinology. 2019;108:256–264. 30352433 10.1159/000494760

[93] Rosenkranz AA Slastnikova TA Karmakova TA. Antitumor activity of Auger electron emitter ^111^In delivered by modular nanotransporter for treatment of bladder cancer with EGFR overexpression. Front Pharmacol. 2018;9:1331. 30510514 10.3389/fphar.2018.01331 PMC6252321

[94] Vandamme T Peeters M Dogan F. Whole-exome characterization of pancreatic neuroendocrine tumor cell lines BON-1 and QGP-1. J Mol Endocrinol. 2015;54:137–147. 25612765 10.1530/JME-14-0304

[95] Scarpa A Chang DK Nones K. Whole-genome landscape of pancreatic neuroendocrine tumours. Nature. 2017;543:65–71. 28199314 10.1038/nature21063

[96] Smith M Parker C Saad F. Addition of radium-223 to abiraterone acetate and prednisone or prednisolone in patients with castration-resistant prostate cancer and bone metastases (ERA 223): a randomised, double-blind, placebo-controlled, phase 3 trial. Lancet Oncol. 2019;20:408–419. 30738780 10.1016/S1470-2045(18)30860-X

[97] Park IH Lee KS Ro J. Effects of second and subsequent lines of chemotherapy for metastatic breast cancer. Clin Breast Cancer. 2015;15:e55–e62. 25445418 10.1016/j.clbc.2014.09.001

